# Antimicrobial resistance of *Enterobacteriaceae* in rabbit farms: an underestimated reservoir harboring *mcr-1.1*

**DOI:** 10.3389/fcimb.2025.1663852

**Published:** 2025-10-01

**Authors:** Zizhe Hu, Dongdong Chen, Tuanyuan Shi, Yee Huang, Xuemei Cui, Xiaoyu Li, Quanan Ji, Guolian Bao, Yan Liu

**Affiliations:** ^1^ Laboratory for Bacterial Diseases of Livestock and Poultry, Institute of Animal Husbandry and Veterinary Medicine, Zhejiang Academy of Agricultural Sciences, Hangzhou, Zhejiang, China; ^2^ Zhejiang A&F University, College of Veterinary Medicine, Hangzhou, China; ^3^ State Key Laboratory for Managing Biotic and Chemical Threats to the Quality and Safety of Agro-products, Institute of Animal Husbandry and Veterinary Sciences, Zhejiang Academy of Agricultural Sciences, Hangzhou, China

**Keywords:** rabbits, *Enterobacteriaceae*, *mcr*-1.1, horizontal transfer, whole genome sequence, surveillance

## Abstract

**Introduction:**

The transmission of antimicrobial resistance (AMR), particularly the antimicrobial resistance gene in *Enterobacteriaceae*, presents a critical challenge to global public health. Sichuan province is the largest producer and consumer of rabbit meat in China. However, few studies have focused on AMR surveillance in rabbits.

**Methods:**

Enterobacteriaceae strains were isolated and identified by MALDI-TOF. The minimum inhibitory concentration (MIC) was determined according to the Clinical and Laboratory Standards Institute. Whole-genome sequencing was performed using the Illumina and Oxford Nanopore Technologies (ONT) platforms.

**Results and discussion:**

A total of 73 *Enterobacteriaceae* strains were isolated, including *Klebsiella pneumoniae*, Salmonella enterica, *Enterobacter hormaechei*, and *Escherichia coli*. Resistance rates to tetracycline, ciprofloxacin, nalidixic acid, sulfamethoxazole-trimethoprim, and ampicillin exceeded 60%. For *Escherichia coli* isolates showed that ST328, ST22, and ST29 were the primary sequence types, with O178:H7 being the predominant serotype. Remarkably, 48% (35/73) of the isolates carried the *mcr-1.1* gene, and among these, 82.9% (29/35) mcr-1.1-positive isolates contained the IncI2 plasmid replicon. The *mcr-1.1* gene in *Klebsiella pneumoniae*, *Salmonella enterica* and *Escherichia coli* transferred to a recipient strain. Furthermore, the genetic environment of the *mcr-1.1* gene showed that it was flanked by *PAP2* and a relaxase. Comparative analysis indicated that the *mcr-1.1*-positive plasmid exhibited high sequence identity to plasmids from human, porcine, and bovine sources. Notably, a phylogenetic analysis based on core single nucleotide polymorphisms demonstrated that certain rabbit-derived *mcr-1*-positive *Escherichia coli* strains clustered within the same evolutionary branch as humanderived strains. These findings indicated that smaller-scale breeding operations, such as rabbit farming, could serve as underrecognized reservoirs of AMR determinants, particularly the *mcr-1.1* gene, thus requiring systematic assessment.

## 1 Introduction

The escalating global emergence of antimicrobial resistance (AMR) stands as one of the most critical public health challenges in the 21st century. By 2050, AMR is projected to cause up to 39.1 million deaths and lead to substantial economic losses (Naghavi et al., 2024). The excessive and inappropriate use of antibiotics in both clinical and agricultural contexts is one of the major factors contributing to the development and spread of AMR (Samreen et al., 2021). Of the diverse resistance mechanisms, the emergence of the plasmid-mediated polymyxin resistance gene *mcr-1* has raised significant concern. Polymyxins are considered the “last line of defense” against multidrug-resistant bacteria, including *Enterobacteriaceae* (Andrade et al., 2020; Mohapatra et al., 2021). The discovery in 2015 of the plasmid mediated *mcr-1* gene marked a significant paradigm shift, as the plasmid facilitated the horizontal gene transfer of polymyxin resistance among bacterial species, thereby posing a substantial threat to the efficacy of this critical antibiotic class (Liu et al., 2016). Alarmingly, the *mcr-1* gene has been detected not only in clinical isolates but also in healthy human carriers and livestock, underscoring its covert and extensive dissemination across various reservoirs beyond traditional healthcare environments (Shen et al., 2018).

The *Enterobacteriaceae* family, which includes the key members *Escherichia coli*, *Klebsiella pneumoniae*, and *Salmonella* spp, is pivotal in the dissemination of the *mcr-1* gene (Xiaomin et al., 2020). These bacteria flourish across a wide range of ecological niches, from the human gut to agricultural environments, with their plasmids serving as vehicles for the spread of antimicrobial resistance genes (ARG) (Castañeda-Barba et al., 2023). Studies have demonstrated that *mcr-1* harboring plasmids, particularly those of the InI2 and IncX4 types, in addition to transposons carrying IS*Apl1*, may contribute to the rapid dissemination of resistance across bacterial populations (Wang et al., 2018). This adaptability is further complicated by the co-integration of *mcr-1* with other resistance determinants, such as *bla*_NDM_ genes, which leads to the emergence of pathogens with dual resistance to colistin and carbapenems (Zhao et al., 2025). The clinical implications are deeply concerning: infections caused by *mcr-1*-positive *Enterobacteriaceae* are linked to prolonged hospital stays, elevated mortality rates, and limited therapeutic options, thereby presenting a significant challenge to global health security (Wang et al., 2017; Naghavi et al., 2024).

The global dissemination of *mcr-1* is not uniform; instead, it is shaped by regional socioecological factors. In China, the Sichuan-Chongqing region is characterized by dense human population centers, intensive livestock systems, and culturally significant dietary practices. This area hosts over 100 million residents and is known for its high consumption of rabbit meat, which accounts for 60% of China’s total consumption. As a result, the region has developed a thriving meat rabbit industry. To meet the increasing demand for rabbit meat, farmers frequently use substantial quantities of antimicrobials during rabbit rearing. Although the widespread use of antibiotics has raised significant concerns within the public health community, the issue remains largely unaddressed in rabbit farming. There have been sporadic studies reporting AMR in rabbit farms (Zhao et al., 2018; Wang et al., 2020a); however, the regions investigated in these studies were not representative of Sichuan province. Most research has concentrated on major livestock industries such as pigs, cattle, sheep, and poultry, with rabbit farming frequently neglected. Intensive farming environments provide ideal conditions for resistance gene proliferation. Furthermore, the absence of standardized AMR monitoring in these settings conceals the true prevalence of ARGs. This gap is particularly significant given Sichuan’s role as a national hub for rabbit meat processing and export, where resistance genes could spread extensively through trade networks. Therefore, it is imperative to investigate the prevalence of AMR in rabbit farms in Sichuan province.

## 2 Material and methods

### 2.1 Samples collection

We collected samples from 10 rabbit farms across Sichuan province for a total of 187 samples. These included swabs from healthy rabbits as well as environmental samples, including anal swabs, nasal swabs, water, feed, cages, feces and sewage. The specific number of samples of each type collected and the corresponding cities are detailed in **Table 1**. To collect the rabbit anal samples, a sterile cotton swab was gently inserted into the rabbit’s anus and carefully rubbed against the rectal mucosa. Subsequently, the swab was placed into a sterilized container containing brain heart infusion medium. To collect environmental samples, a swab was moistened in brain heart infusion medium in a tube. Next, the environmental surfaces were systematically swabbed, including cages, feed troughs, and floors, ensuring thorough coverage. Finally, the swab was returned to the tube.

**Table 1:** Distribution of sampled rabbit farms and bacterial isolates by geographic locality.

Locality	Sample type	Number of farms	Number of samples	Isolates
Chengdu city	Anal swabs, nasal swabs, cages	1	7	4
Leshan city	Anal swabs, nasal swabs, floor	1	10	3
Rongxian county	Anal swabs, nasal swabs, water, feed, cages, feces, sewage	4	65	29
Zigong city	Anal swabs, nasal swabs, water, feed, cages, feces, sewage	4	105	37
Total	**-**	**10**	**187**	**73**

samples were collected from meat rabbit farms in Sichuan province, China. The samples included both animal and environmental specimens. The bold values represent the total of each column.

### 2.2 *Enterobacteriaceae* isolation and identification

The collected samples were pre-cultured in an incubator at 37°C, after which a single loop of each bacterial solution was streaked on a MacConkey agar plate. The plates were cultured in an incubator at 37°C for 24 h. Then, single pink clones were selected and incubated on trypticase soy agar plates. A pure and single colony was carefully aspirated with a sterile pipette tip and gently deposited onto a clean MALDI target plate to form a thin even layer. Subsequently, 1 μL of α-cyano-4-hydroxycinnamic acid matrix solution was accurately dispensed onto each spotted sample and allowed to dry naturally at room temperature. The MALDI target plate was then immediately transferred to a MALDI-TOF mass spectrometer (Bruker) for isolate identification. To avoid strain duplication, a single representative strain was retained from each sample.

### 2.3 Antimicrobial susceptibility test

The minimum inhibitory concentration (MIC) was determined according to the Clinical and Laboratory Standards Institute (CLSI) M100-33rd guidelines using 96-well plates to test the 17 antimicrobials: nalidixic acid, ciprofloxacin, colistin, tigecycline, tetracycline, chloramphenicol, azithromycin, trimethoprim-sulfamethoxazole, amikacin, streptomycin, ampicillin, cefotaxime, ceftazidime, ceftazidime-avibactam, ampicillin-sulbactam, meropenem, and ertapenem. Single and pure isolated colonies were picked to prepare a 0.5 McFarland standard bacterial suspension. Subsequently, the bacterial suspension was diluted 100-fold using Müller–Hinton broth. Then, the bacterial suspension was added to a 96-well plate manufactured by Meihua Company (China) that had been preloaded with concentration gradients of antimicrobial drugs. The *Escherichia coli* ATCC^®^ 25922 reference strain served as the quality control strain throughout the study. MIC determinations were conducted in triplicate for each clinical isolate. The plates were incubated at 37°C for 18–20 h. The resistant phenotype of the isolates was determined according to the MIC breakpoint criteria outlined in the CLSI M100-33rd guidelines.

### 2.4 Conjugation assay

In the conjugation assay, Ec-A21, Ec-A24, Ec-A29, Ec-JB2, and Ec-CD45 isolates that exhibited colistin MIC values exceeding 4 mg/L served as donor strains, while *E. coli* J53 functioned as the recipient strain. Donor and recipient strains were cultured to logarithmic growth and subsequently mixed at a 1:1 volumetric ratio (0.4 mL of the donor and 0.4 mL of the recipient). Following static incubation for 10 min, 80 μL of the bacterial suspension (40 μL of the donor and 40 μL of the recipient) was aseptically transferred onto sterile 0.22-μm nitrocellulose membranes placed on trypticase soy agar plates and incubated for 12 h at 37 °C. Post-incubation, all cultures (donors, recipients, and conjugation mixtures) were washed with phosphate-buffered saline. Transconjugant selection was performed using Müller–Hinton agar supplemented with 100 mg/L sodium azide and 4 mg/L colistin. Donor viability was quantified by plating serial dilutions on sodium azide-containing agar (100 mg/L). PCR amplification of *mcr-1* (primer sequences, F: 5'-CGG TCA GTC CGT TTG TTC-3' and R: 5'-CTT GGT CGG TCT GTA GGG-3') was performed (Liu et al., 2016). The conjugation transfer frequency was calculated by dividing the number of transconjugants by the number of recipients.

### 2.5 Whole-genome sequencing and genome assembly

Whole-genome sequencing of the isolated strains was conducted by Majorbio Bio-pharm Technology (China) using the Illumina platform. The sequencing procedure was as follows: Initially, total genomic DNA was extracted from the isolated strains using a bacterial genomic DNA extraction kit. The extracted genomic DNA was then fragmented using Covaris technology, and a genomic sequencing library was constructed. Draft genomes were generated on the Illumina sequencing platform. Sequencing libraries with insert sizes of approximately 400 bp were constructed using only DNA samples that met stringent quality control standards. The libraries were subsequently subjected to paired-end sequencing with a read length of 150 bp in each direction. This process generated raw sequencing data with a minimum coverage depth of 100× across the genome. SOAPdenovo 2.04 software was used for genome assembly, leading to the construction of multiple scaffolds.

Three *E. coli* complete genomes were obtained using an Oxford Nanopore Technologies (ONT) system in combination with Illumina genome data. This was performed by Biomaker Technology Company (China). The experimental procedure was conducted in accordance with the standard protocol provided by ONT, which includes sample quality assessment, library preparation, library quality evaluation, and sequencing. The main steps were as follows: high-quality genomic DNA was extracted using bacterial genome extraction kits and subsequently assessed for purity, concentration, and integrity using Nanodrop, Qubit, and 0.35% agarose gel electrophoresis; large DNA fragments were size-selected and recovered using the BluePippin fully automated nucleic acid recovery system; library construction was carried out using the SQK-LSK109 ligation kit, followed by sequencing. To assemble the genome, the filtered reads were first assembled using Canu v1.5 software, followed by circularization of the assembled genome using Circlator v1.5.5. For functional annotation, the predicted proteins were compared against the Nr, Swiss-Prot, TrEMBL, KEGG, and eggNOG databases using BLAST with an e-value threshold of 1e−5. The *Escherichia coli* strains Ec-JB2 and Ec-CD45 were subjected to whole-genome sequencing using ONT sequencing system. Both strains contained five plasmids, with plasmids pEc-JB2-5 (GenBank accession: CP182207) and pEc-CD45-5 (GenBank accession: CP182224) of particular interest as they carried the *mcr-1* colistin resistance gene.

### 2.6 Bioinformatic analysis

Isolate identification was validated using conserved housekeeping genes via the Majorbio cloud platform (Ren et al., 2022). Subsequently, the genomes were uploaded to KmerFinder 3.2 (https://cge.food.dtu.dk/services/KmerFinder/) and subjected to BLAST analysis to identify isolates. ResFinder 4.7.2 (http://genepi.food.dtu.dk/resfinder) and the Comprehensive Antibiotic Resistance Database (https://card.mcmaster.ca/) was used to predict ARGs, and the Virulence Factor Database (Liu et al., 2022) was used to predict virulence factor genes (VFG). Both analyses used a BLAST nucleotide identity threshold of ≥90% and length coverage ≥90%. The Center of Genome Epidemiology MLST 2.0 tool (https://cge.food.dtu.dk/services/MLST/) was used to predict the sequence types (ST) of the isolates. Pathogenwatch (https://pathogen.watch/) was used for *Klebsiella pneumoniae* and *Salmonella enterica* serotype prediction, and ClermonTyping (http://clermontyping.iame-research.center/) was used to predict *E. coli* phylogroups. Proksee (https://proksee.ca/) was used to annotate resistant plasmids, the genome sequences were annotated using Prokka, and mobileOG-db (beatrix-1.6) was used to find mobile genetic elements (MGE) (Seemann, 2014; Brown et al., 2022). BacWGSTdb (http://bacdb.cn/BacWGSTdb/index.php) was utilized for phylogenetic analysis of *E. coli* based on core single nucleotide polymorphisms (SNP) (Feng et al., 2021), with *E. coli* MG1655 selected as the reference genome.

### 2.7 Data visualization

TBtools v2.210 was used to generate heatmaps of the ARGs and VFGs (Chen et al., 2023). Office 2021 Excel was used to collect and process data in tables. BLAST Ring Image Generator (BRIG) V0.95 was used to analyze the resistant plasmid homology. NCBI BLAST v2.16.0 was used for the local alignment of plasmid sequences. GraphPad Prism 8.0 was used to create column charts. Evolview 2.0 was used to modify the phylogenetic tree (He et al., 2016). Proksee was used to visualize the map of resistant plasmids (Grant et al., 2023).

### 2.8 Data availability

All genome sequences were uploaded to NCBI and whole-genome shotgun data was deposited in GenBank under at Bioproject PRJNA1223317. Data will be made available on request.

## 3 Results

### 3.1 Isolation and identification of *Enterobacteriaceae*

A total of 187 samples were collected from four cities in Sichuan province, China (Chengdu, Leshan, Rongxian, and Zigong), originating from ten rabbit farms. The samples included anal swabs, nasal swabs, feces, water, feed, floor, cages, and sewage. A total of 73 *Enterobacteriaceae* strains were isolated using MacConkey selective culture and MALDI-TOF mass spectrometry, comprising 3 *Klebsiella pneumoniae* strains, 3 *Salmonella enterica* strains, 6 *Enterobacter hormaechei* strains, and 61 *Escherichia coli* strains. Detailed information on the collected samples and isolates is provided in **Table 1**.

### 3.2 Antimicrobial susceptibility profiles and resistance patterns

**Figure 1:**
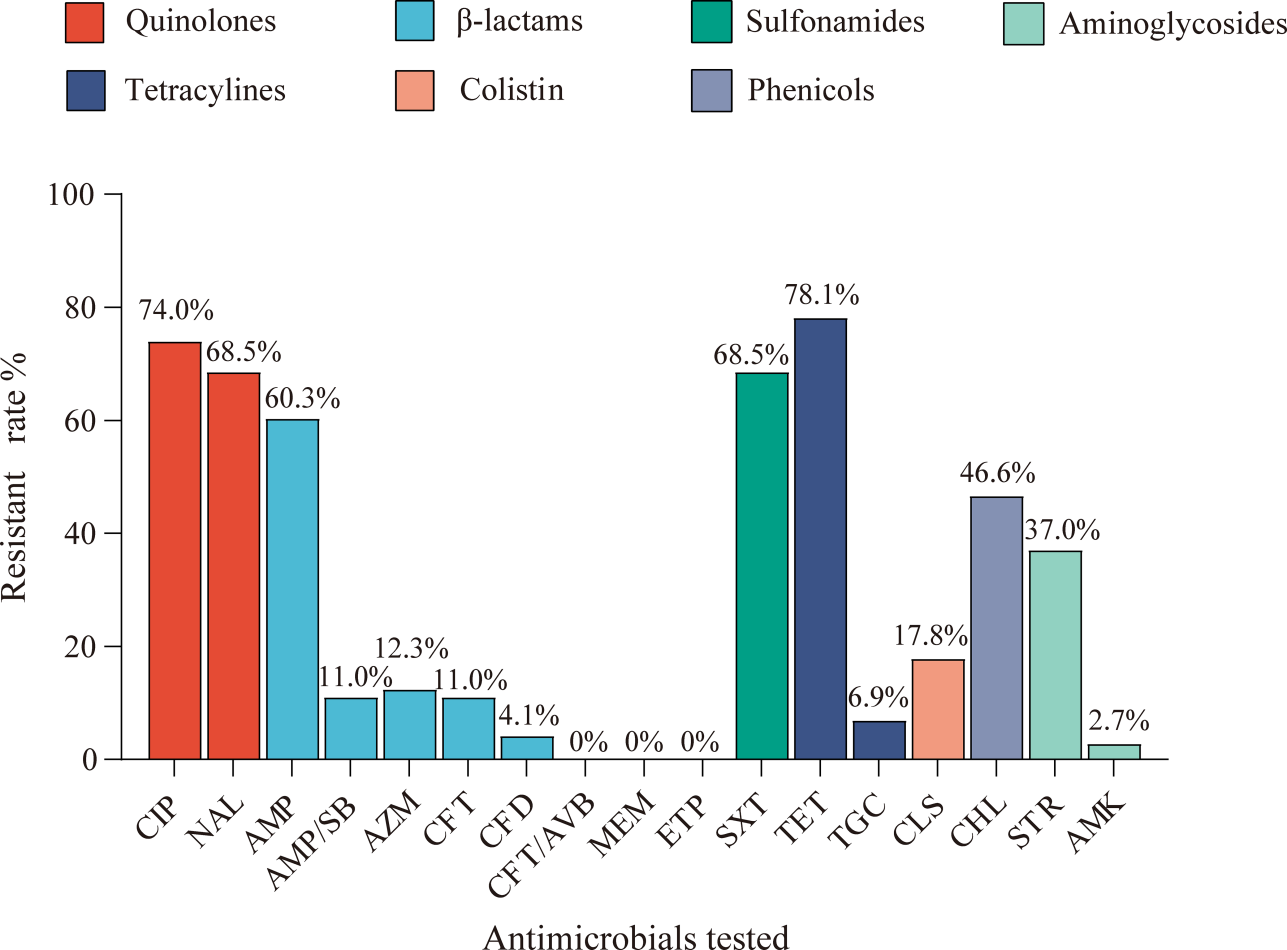
The resistance rates of 17 antimicrobial agents among the tested isolates in rabbits. The resistance rates of 73 strains of *Enterobacteriaceae* isolates against ciprofloxacin (CIP), nalidixic acid (NAL), ampicillin (AMP), ampicillin/sulbactam (AMP/SB), azithromycin (AZM), cefotaxime (CFT), ceftazidime (CFD), ceftazidime-avibactam (CFT/AVB), meropenem (MEM), ertapenem (ETP), sulfamethoxazole/trimethoprim (SXT), tetracycline (TET), tigecycline (TGC), colistin (CLS), chloramphenicol (CHL), streptomycin (STR), and amikacin (AMK), in which the colors represent seven categories of antimicrobials.

Antimicrobial susceptibility profiles of the isolates were determined against 17 antimicrobials spanning seven therapeutic classes. As depicted in **Figure 1**, five antimicrobials demonstrated resistance rates exceeding 60%: tetracycline (78%, 57/73), ciprofloxacin (74%, 54/73), nalidixic acid (68.5%, 50/73), sulfamethoxazole-trimethoprim (68.5%, 50/73), and ampicillin (60.3%, 44/73). Moderate resistance was observed for chloramphenicol (46.6%, 34/73) and streptomycin (37%, 27/73). Notably, emerging resistance to last-line antibiotics was detected, with 17.8% (13/73) of isolates demonstrating colistin resistance and 6.9% (5/73) showing reduced tigecycline susceptibility. In addition, the resistance rates to cefotaxime, ceftazidime, and ampicillin-sulbactam were 11% (8/73), 4.1% (3/73), and 11% (8/73), respectively, suggesting that these isolates may include Extended-spectrum β-lactamase (ESBL)-producing strains. Azithromycin resistance occurred in 12.3% (9/73) of strains, while two isolates exhibited resistance to amikacin. Importantly, all isolates were susceptible to carbapenems (meropenem and ertapenem) and the novel β-lactamase inhibitor combination ceftazidime-avibactam. Multidrug resistance (MDR), defined as resistance to ≥3 antimicrobials, was observed in 86.3% (63/73) of isolates (**Supplementary Figure S1**). The antimicrobial resistance profiles of the isolates are summarized in **Supplementary Table S1**, which indicates that certain individual isolates demonstrate resistance to as many as 11 antimicrobial agents. One isolate exhibited pan-susceptibility, while the majority of isolates (75.3%, 55/73) displayed resistance to 3–7 antimicrobial classes. **Supplementary Table S1** shows the MICs and resistance profiles of all isolates.

### 3.3 Molecular characterization of isolates

Multilocus sequence typing of the 61 *Escherichia coli* isolates identified 16 distinct sequence types, with three predominant clones collectively representing 52.4% of the population: ST328 (26.2%, 16/61), ST224 (13.1%, 8/61), and ST297 (13.1%, 8/61). **Supplementary Table S2** shows all isolate STs. Serological profiling revealed 22 unique O:H serovars, including four strains (6.6%) with unknown O antigens. **Supplementary Table S3** shows all *E. coli* serotype alignment results. The most prevalent serovars were O178:H7 (19.7%, 12/61), O1:H10 (9.8%, 6/61), and O172:H3 (9.8%, 6/61). Using phylogenetic grouping analysis, the isolates were classified into five phylogroups: A, B1, B2, C, and E, with the majority belonging to phylogroup B1 (78.7%, 48/61). Published reference has reported that B1 strains are predominant in domestic and wild animals (Tenaillon et al., 2010). **Supplementary Table S4** shows all *E. coli* phylogroups alignment results. All three strains of *Salmonella enterica* isolate belonged to ST426, with a serotype classification of Aberdeen. The *Klebsiella pneumoniae* collection (*n* = 3) included two STs: ST1876 (*n* = 2) and ST294 (*n* = 1), with serotyping identifying one O1ab:K30 strain. The remaining two isolates displayed O13 serotype compatibility, with the H serotype not classified, indicating a possible capsular antigenic variation or genetic deletion in the K locus. The phenotypes predicted from the isolates’ genomic data are presented in detail in **Table 2**.

**Table 2:** The characteristic information of 73 isolates from rabbit farms located in Sichuan province.

Isolates	Identification	Accession number	Sequence type	Serotype	Phylogroup
Ec-A21	*Klebsiella pneumoniae*	JBLRDQ000000000	ST1876	O13:NC	NC
Ec-A24	*Klebsiella pneumoniae*	JBLRDP000000000	ST1876	O13:NC	NC
Ec-A31	*Klebsiella pneumoniae*	JBLRDO000000000	ST294	O1ab:K30	NC
Ec-A29	*Salmonella enterica*	JBLRDN000000000	ST426	Aberdeen	NC
Ec-B30	*Salmonella enterica*	JBLRDM000000000	ST426	Aberdeen	NC
Ec-C	*Salmonella enterica*	JBLRDL000000000	ST426	Aberdeen	NC
Ec-A	*Enterobacter hormaechei*	JBLRDK000000000	ST693	NC	NC
Ec-B29	*Enterobacter hormaechei*	JBLRDJ000000000	ST419	NC	NC
Ec-B31	*Enterobacter hormaechei*	JBLRDI000000000	Unknown	NC	NC
Ec-B37	*Enterobacter hormaechei*	JBLRDH000000000	ST1683	NC	NC
Ec-D3	*Enterobacter hormaechei*	JBLRDG000000000	ST3371	NC	NC
Ec-D4	*Enterobacter hormaechei*	JBLTWX000000000	ST1131	NC	NC
Ec-A1	*Escherichia coli*	JBLRCM000000000	ST297	O86:H49	E
Ec-A15	*Escherichia coli*	JBLRCG000000000	ST707	O84:H23	A
Ec-A4	*Escherichia coli*	JBLRCL000000000	ST297	O86:H49	E
Ec-A6	*Escherichia coli*	JBLRCK000000000	ST297	O1:H10	B1
Ec-A7	*Escherichia coli*	JBLRCJ000000000	ST707	O84:H23	A
Ec-A8	*Escherichia coli*	JBLRCI000000000	ST16119	O175:H28	B1
Ec-A9	*Escherichia coli*	JBLRCH000000000	ST224	O172:H23	B1
Ec-B	*Escherichia coli*	JBLRCF000000000	ST224	O163:H23	B1
Ec-B10	*Escherichia coli*	JBLRCA000000000	ST297	O1:H10	B1
Ec-B11	*Escherichia coli*	JBLRBZ000000000	ST297	O1:H10	B1
Ec-B12	*Escherichia coli*	JBLRBY000000000	Unknown	NC:H5	A
Ec-B2	*Escherichia coli*	JBLRCE000000000	ST20	O145:H2	B1
Ec-B22	*Escherichia coli*	JBLRBX000000000	ST297	O1:H10	B1
Ec-B33	*Escherichia coli*	JBLRBW000000000	ST180	O156:H7	B1
Ec-B34	*Escherichia coli*	JBLRBV000000000	ST2448	O103:H7	B1
Ec-B5	*Escherichia coli*	JBLRCD000000000	ST707	O84:H23	A
Ec-B7	*Escherichia coli*	JBLRCC000000000	ST297	O1:H10	B1
Ec-B8	*Escherichia coli*	JBLRCB000000000	ST297	O1:H10	B1
Ec-C1	*Escherichia coli*	JBLRBU000000000	ST75	NC:H8	B1
Ec-C12	*Escherichia coli*	JBLRBQ000000000	ST224	O172:H23	B1
Ec-C16	*Escherichia coli*	JBLRBP000000000	ST155	O184:H51	B1
Ec-C17	*Escherichia coli*	JBLRBO000000000	ST3558	O148:H8	B1
Ec-C18	*Escherichia coli*	JBLRBN000000000	ST1431	O8:H19	B1
Ec-C2	*Escherichia coli*	JBLRBT000000000	ST707	O84:H23	A
Ec-C21	*Escherichia coli*	JBLRBM000000000	ST224	O172:H23	B1
Ec-C5	*Escherichia coli*	JBLRBS000000000	ST224	O172:H23	B1
Ec-C7	*Escherichia coli*	JBLRBR000000000	ST224	O172:H23	B1
Ec-D	*Escherichia coli*	JBLRBL000000000	ST141	O50:H6	B2
Ec-D13	*Escherichia coli*	JBLRBK000000000	ST141	O50:H6	B2
Ec-D14	*Escherichia coli*	JBLRBJ000000000	ST224	O172:H23	B1
Ec-D15	*Escherichia coli*	JBLRBI000000000	ST156	NC:H10	B1
Ec-D16	*Escherichia coli*	JBLRBH000000000	ST328	O153:H7	B1
Ec-D17	*Escherichia coli*	JBLRBG000000000	ST141	O50:H6	B2
Ec-D18	*Escherichia coli*	JBLRBF000000000	Unknown	O167:H14	B1
Ec-D20	*Escherichia coli*	JBLRBE000000000	Unknown	O178:H7	B1
Ec-D21	*Escherichia coli*	JBLRBD000000000	ST4380	O96:H23	B1
Ec-D23	*Escherichia coli*	JBLRBC000000000	ST141	O50:H6	B2
Ec-D24	*Escherichia coli*	JBLRBB000000000	ST88	O8:H11	C
Ec-D26	*Escherichia coli*	JBLRBA000000000	ST141	O50:H6	B2
Ec-CD44	*Escherichia coli*	JBLRCP000000000	ST14383	O18ac:H7	B1
Ec-CD45	*Escherichia coli*	CP182220-CP182224	ST328	O153:H7	B1
Ec-CD47	*Escherichia coli*	JBLRCO000000000	ST328	O153:H7	B1
Ec-CD55	*Escherichia coli*	JBLRCN000000000	ST328	O153:H7	B1
Ec-JB1	*Escherichia coli*	JBLRDF000000000	ST20	O128ac:H2	B1
Ec-JB2	*Escherichia coli*	CP182203-CP182207	ST20	O128ac:H2	B1
Ec-JB3	*Escherichia coli*	JBLRDE000000000	ST328	O178:H7	B1
Ec-RX11	*Escherichia coli*	JBLRDD000000000	ST328	O178:H7	B1
Ec-RX13	*Escherichia coli*	JBLRDC000000000	ST224	O78:H23	B1
Ec-RX15	*Escherichia coli*	JBLRDB000000000	ST20	O128ac:H2	B1
Ec-RX16	*Escherichia coli*	JBLRDA000000000	ST328	O178:H7	B1
Ec-RX18	*Escherichia coli*	JBLRCZ000000000	ST328	O178:H7	B1
Ec-RX19	*Escherichia coli*	JBLRCY000000000	ST162	O9:H19	B1
Ec-RX24	*Escherichia coli*	CP182136-CP182138	Unknown	NC:H16	B1
Ec-RX28	*Escherichia coli*	JBLRCX000000000	ST328	O178:H7	B1
Ec-RX38	*Escherichia coli*	JBLRCW000000000	ST328	O178:H7	B1
Ec-RX39	*Escherichia coli*	JBLRCV000000000	ST328	O153:H7	B1
Ec-RX41	*Escherichia coli*	JBLRCU000000000	ST328	O178:H7	B1
Ec-RX42	*Escherichia coli*	JBLRCT000000000	ST328	O178:H7	B1
Ec-RX49	*Escherichia coli*	JBLRCS000000000	ST328	O178:H7	B1
Ec-RX50	*Escherichia coli*	JBLRCR000000000	ST328	O178:H7	B1
Ec-RX51	*Escherichia coli*	JBLRCQ000000000	ST328	O178:H7	B1

NC means not classified.

### 3.4 ARG, VFG and plasmid replicon analyses

**Figure 2:**
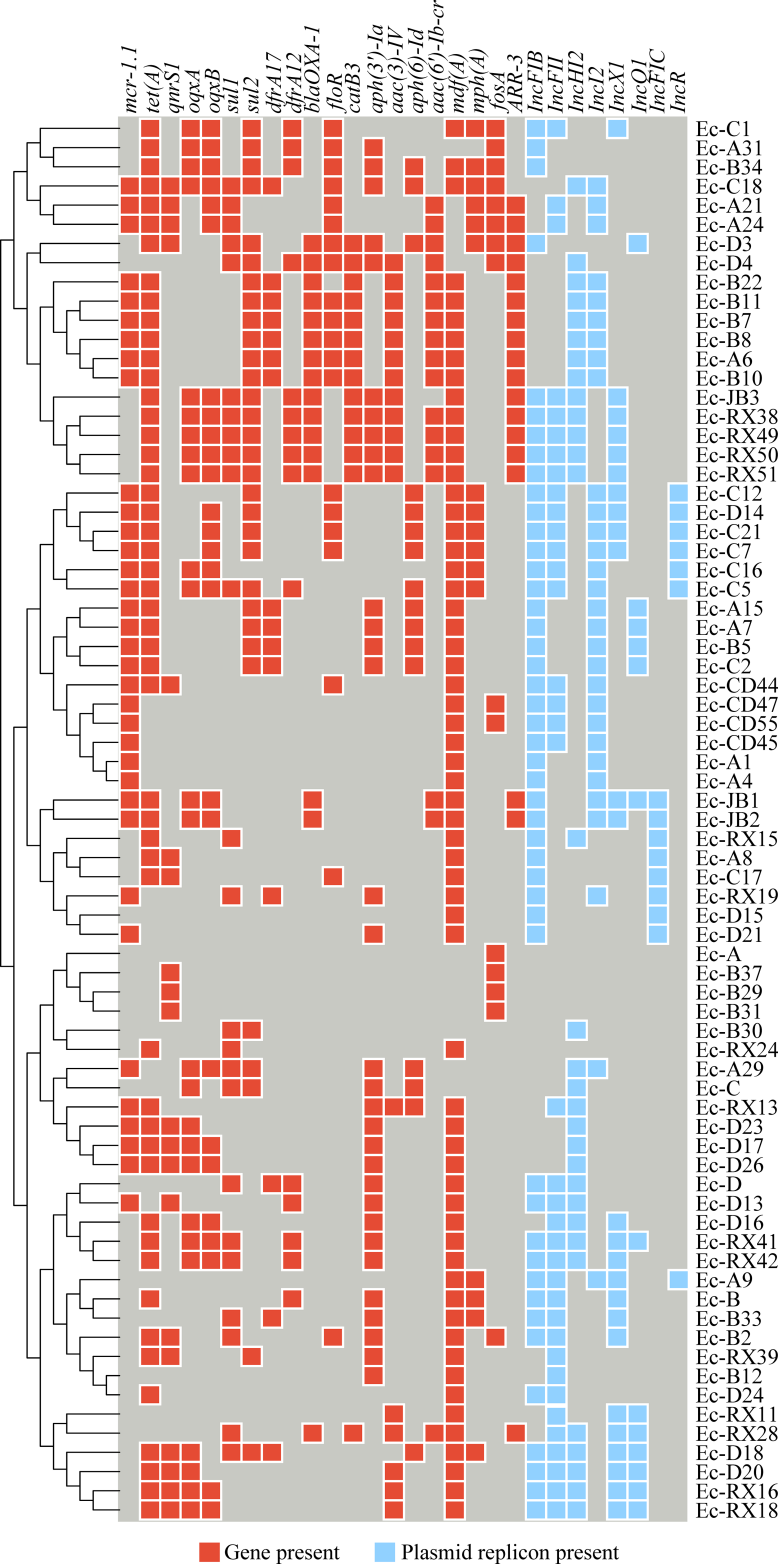
A heat map illustrates the distribution of antimicrobial resistance genes (ARG) and plasmid replicon types among the isolates. Red cells indicate the presence of an ARG, light blue cells indicate the presence of a plasmid replicon, and deep blue cells indicate that neither is present.

The ARGs identified in the *Enterobacteriaceae* strains included the following types of resistance: polymyxin resistance (*mcr-1.1*), tetracycline resistance (*tet(A)*), fluoroquinolone resistance (*qnrS1, oqxA, oqxB*), sulfonamide and diaminopyrimidine resistance (*sul1, sul2, dfrA12, dfrA17*), extended-spectrum β-lactamase production (*bla*_CTX-M_), chloramphenicol resistance (*catB3, folR*), aminoglycoside resistance (*aac(3)-IV, aac(6’)-Ib-cr, aph(3’)-Ia, aph(6)-Id*), macrolide resistance (*mph(A)*), and fosfomycin resistance (*fosA*). A subset of the detailed results is presented in **Figure 2**, with Resfinder database annotations summarized in **Supplementary Table S5** and CARD database annotations in **Supplementary Table S6**. The plasmid replicon predictions found that IncFIB, IncFII, IncHI2, Incl2, and IncX1 were the primary replicon types. The annotated results are shown in **Supplementary Table S7**. As shown in **Figure 2**, the *mcr-1.1* gene was detected in 35 isolates; among these, 29 isolates harbored the IncI2 plasmid replicon.

The VFDB database was used to annotate virulence genes encompassed: *vgrG/tssl, espL1, fimC, fimD, fimH, cgsG, csgA, csgC, yagW/ecpD, ompA, clbK, clbJ, cheY, phoP, rcsB, rpoS, gndA, pic, escV* and *iroN*, which associated with *E. coli* adherence, invasion, effector delivery system, exotoxin, motility, and regulation function. The carriage status of major VFGs for each isolated strain was presented in **Supplementary Figure S2**, while the original annotation data are provided in **Supplementary Table S8**.

### 3.5 *mcr-1.1* carried plasmid genetic construct and conjugation experiment

**Figure 3:**
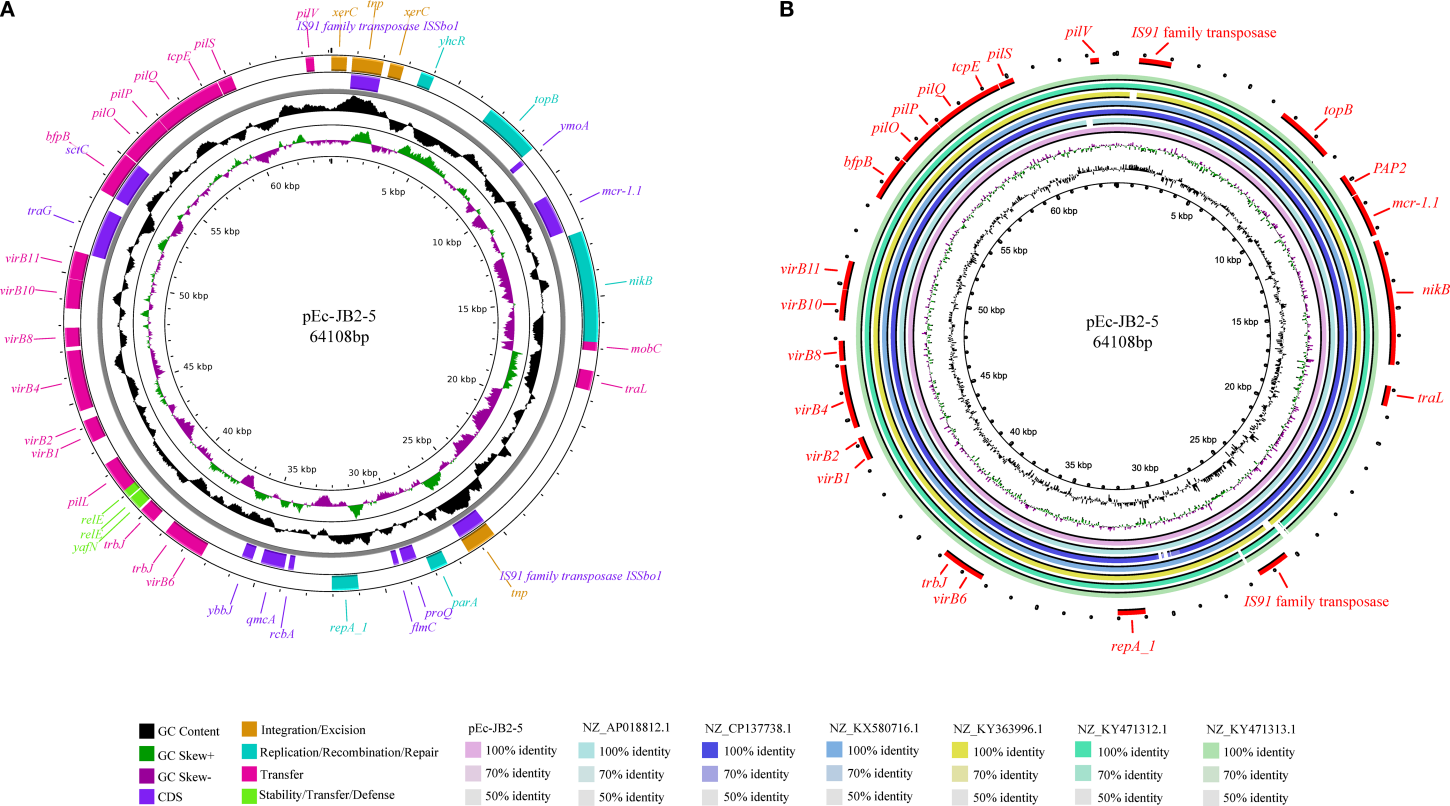
Map and homology analysis of the *mcr-1*-positive plasmid pEc-JB2-5. **(A)** The map of pEc-JB2–5 is presented, in which the rings from inside to outside represent GC content; GC skew; coding sequences; integration and excision regions; replication, recombination, and repair functions; transfer mechanisms; and stability, transfer, and defense modules. **(B)** Homology analysis of the pEc-JB2–5 plasmid is shown, with the rings from inside to outside representing pEc-JB2-5, pE2865-4 (NZ_AP018812.1), an unnamed plasmid from HNSQ2209 (NZ_CP137738.1), an unnamed plasmid from ZJ1635 (NZ_KX580716.1), pSh487-m4 (NZ_KY363996.1), pMCR-M19242 (NZ_KY471312.1), and pMCR-M19441 (NZ_KY471313.1). The outermost red ring represents coding genes.

Plasmid pEc-JB2–5 is 64,108 bp in length and belongs to the IncI2 replicon type. Plasmid pEc-CD45–5 is 80,958 bp in length and also belongs to the IncI2 replicon type. Both plasmids harbor an extensive array of mobile genetic elements, which are organized into four functional clusters: integration and excision elements (*tnp*, *xerC*); replication, recombination, and repair systems (*nikB*, *topB*, *yhcR*, *parA*, *repA-1*); conjugative transfer apparatus (*virB1*–*virB11* operon); and plasmid stability and defense mechanisms (*relE* toxin-antitoxin system, plasmid conjugative transfer pilus *pilP*–*pilQ*, and *tcpE*). Notably, the *mcr-1.1* gene was positioned between a *PAP2* family hydrolase gene and the *nikB* relaxase, a critical enzyme mediating plasmid conjugation through single-strand DNA processing. This genetic architecture, in which antibiotic resistance determinants were flanked by conjugation-associated elements, suggested that it may enhance horizontal dissemination. Plasmid pEc-JB2–5 architecture and MGE organization are schematically depicted in **Figure 3A**, with distinct color-coding to differentiate functional modules. A comparative genomic analysis revealed that pEc-JB2–5 exhibited similarity with clinically relevant plasmids from various host species (**Figure 3B**), nucleotide identity threshold of ≥90% and length coverage ≥90%. These plasmids include pE2865-4 (origin: cattle; geographic location: Japan; size: 62,235 bp; accession number: NZ_AP018812.1); an unnamed plasmid (origin: pig; geographic location: Henan, China; size: 142,379 bp; accession number: NZ_CP137738.1); pMCR-M19242 (origin: human; geographic location: Canada; size: 61,632 bp; accession number: NZ_KY471312.1); Sh487-m4 (origin: human; geographic location: Shanghai, China; size: 63,512 bp; accession number: NZ_KY363996.1); and an unnamed nosocomial infection-associated plasmid (origin: human; geographic location: China; size: 62,440 bp; accession number: NZ_KX580716.1). The pEc-CD45–5 plasmid was also analyzed in the same method, with the detailed results shown in **Supplementary Figure S3**. pEc-CD45–5 exhibited homology with pPSS-08-2_3(origin: human; geographic location: Ecuador; size: 60,961 bp; accession number: NZ_AP027682.1), pPSS-16_2 (origin: human; geographic location: Ecuador; size: 60,960 bp; accession number: NZ_AP027715.1), pHLJ109-70 (origin: chicken; geographic location: China; size: 61,023 bp; accession number: NZ_MN232201.1), pHLJ111-18 (origin: chicken; geographic location: China; size: 60,962 bp; accession number: NZ_MN232205.1), pHLJ111-5 (origin: chicken; geographic location: China; size: 61,094 bp; accession number: NZ_MN232208.1), and pSC111 (origin: human; geographic location: China; size: 60,960 bp; accession number: NZ_MZ277864.1)The cross-species homology and transcontinental distribution of these plasmids, spanning cattle, swine, and human hosts, suggested that pEc-JB2–5 may represent a high-risk mobile genetic element. This finding again highlighted the potential for interspecies transmission of colistin resistance determinants within “One Health” ecosystems.

**Figure 4:**
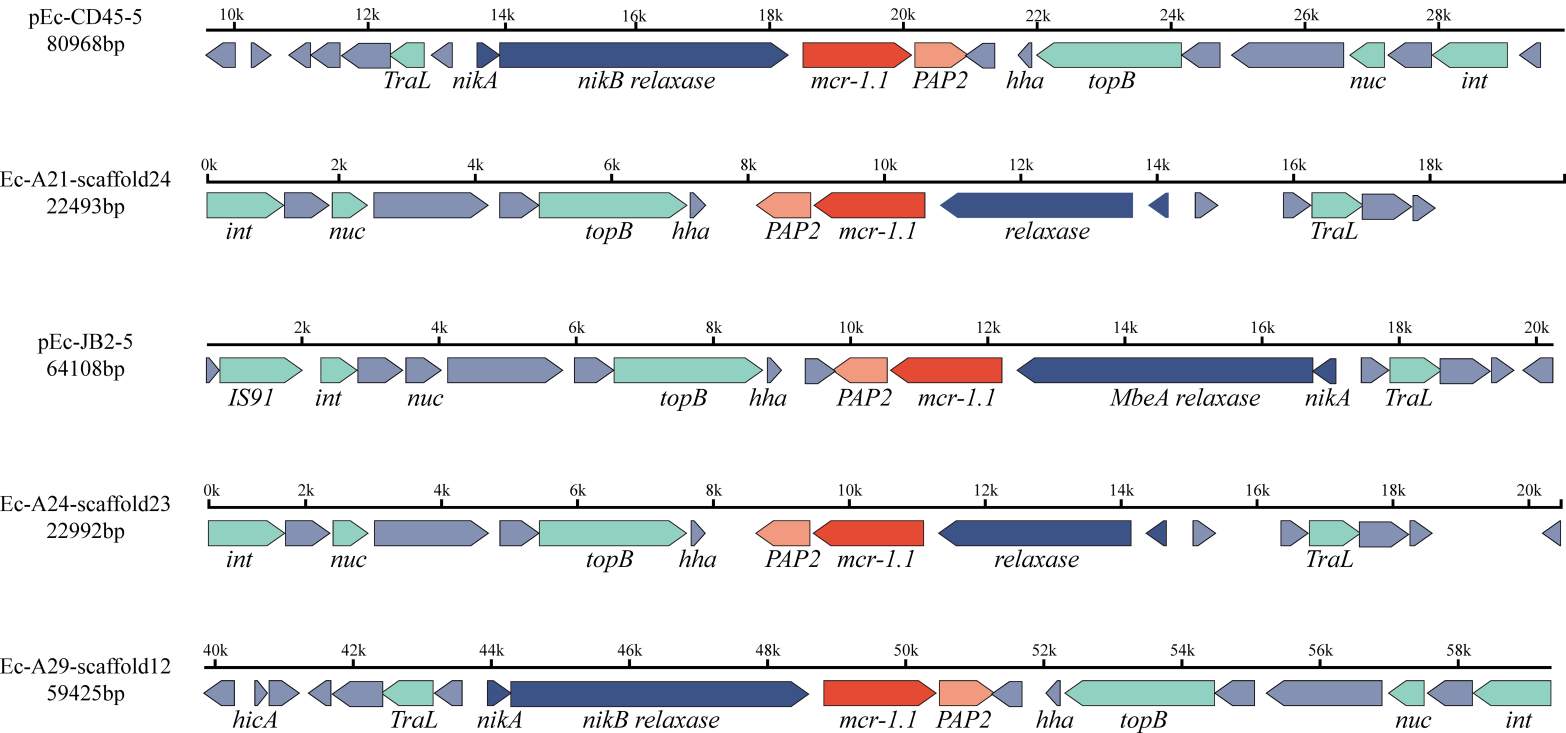
The genetic environment of the *mcr-1.1* gene in five isolates, identified as *Escherichia coli* (Ec-JB2, Ec-CD45), *Klebsiella pneumoniae* (Ec-A21, Ec-A24), and *Salmonella enterica* (Ec-A29). The direction of the arrow indicates the direction of gene coding. Different colors represent proteins with distinct functions: red arrows indicate antimicrobial resistance genes, blue arrows represent relaxases, light green arrows correspond to mobile genetic effectors, and gray arrows denote hypothetical proteins. The *mcr-1.1* gene is flanked by *PAP2* and a relaxase.

As shown in **Figure 2**, the strains Ec-JB2, Ec-CD45, Ec-A21, Ec-A24, and Ec-A29, identified as *Escherichia coli, Klebsiella pneumoniae*, and *Salmonella enterica*, harbored the *mcr-1.1* gene. These isolates harbored multiple plasmid replicons: Ec-JB2 carried IncFIB, IncFIC, IncI1-I(Alpha), IncI2, and IncX1; Ec-CD45 carried IncFIB, IncFII, IncI2; Ec-A21 carried IncI2 and IncFII; Ec-A24 carried IncI2, IncFII, and repB; and Ec-A29 carried IncI2 and IncHI2. All of them contained the IncI2 plasmid replicon. Detailed information on the specific plasmids is provided in Supplementary Material **Supplementary Table S7**. A conjugation experiment demonstrated that all five strains were able to transfer the *mcr-1.1* gene to a recipient strain, *E. coli* J53. The conjugation transfer frequency was performed in **Supplementary Table S9**. The genetic environments of *mcr-1.1* shown in **Figure 4**. Amplification of the *mcr-1* gene in donors, recipients, and transconjugants is illustrated in **Supplementary Figure S4**. As depicted in **Figure 4**, both copies of the *mcr-1* gene were flanked by a relaxase and *PAP2*.

### 3.6 Phylogenetic relationship between rabbit and human source *mcr-1.1 E. coli*

**Figure 5:**
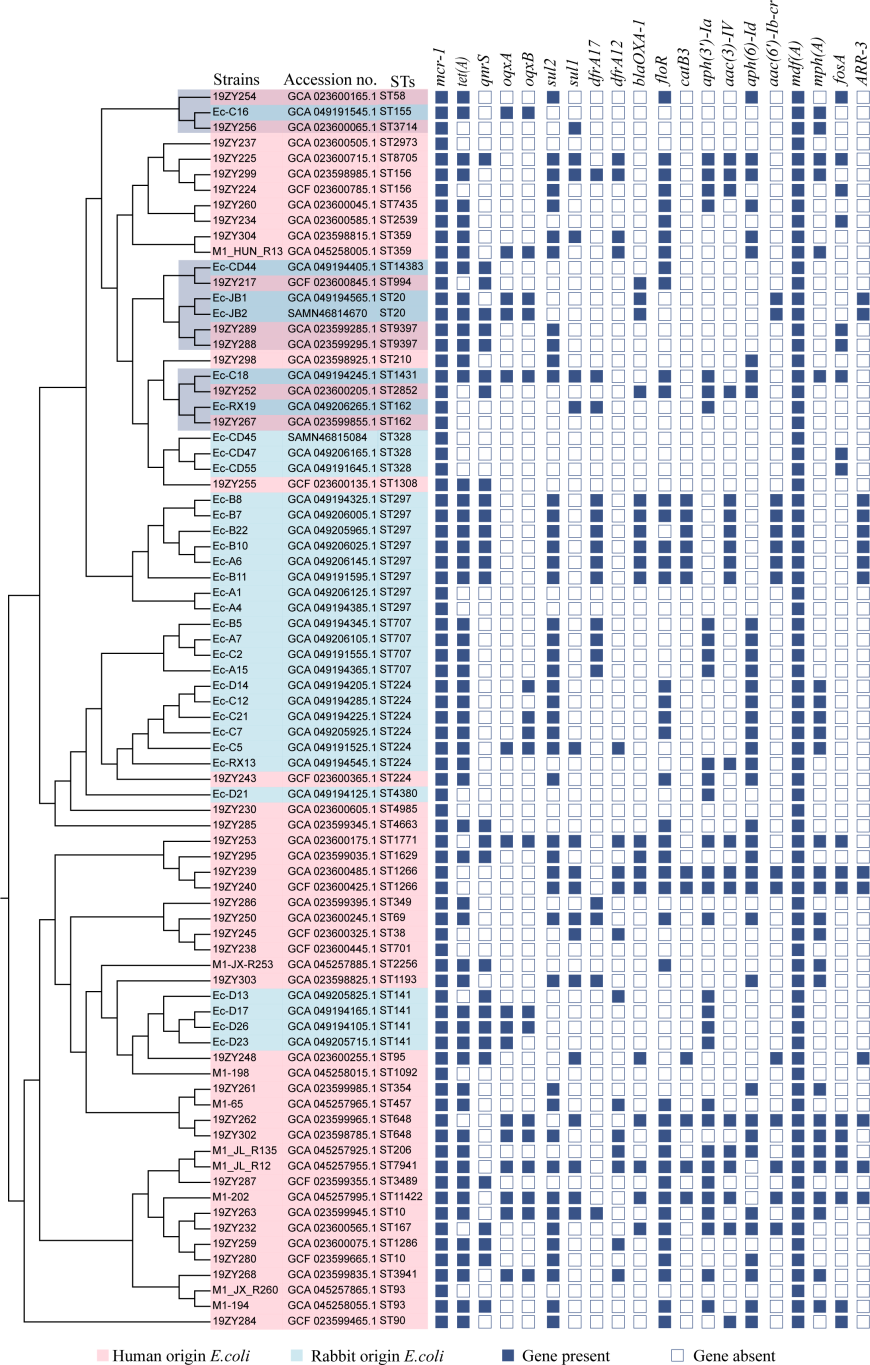
A phylogenetic evolutionary tree was constructed based on core single nucleotide polymorphisms. Pink leaves indicate *E. coli* isolates from humans, and light blue leaves represent *mcr-1*-positive *E. coli* isolates from rabbits in this study. Antimicrobial resistance genes are displayed as blue rectangular cells, in which solid cells denote the presence of the specified gene, and empty cells indicate the absence of the gene.

Whole-genome sequences of 48 human-derived *mcr-1*-carrying *Escherichia coli* strains were systematically retrieved from the NCBI database. These were subjected to comparative phylogenetic analysis with 32 of the rabbit-derived *mcr-1*-harboring *E. coli* strains investigated in the current study. The resulting phylogenetic reconstruction demonstrated some rabbit-origin *Escherichia coli* carrying *mcr-1* and human-origin *Escherichia coli* are on the same evolutionary branch, as marked by the shaded area in **Figure 5**. The phylogenetic clusters derived from human reservoirs are highlighted in pink, while strains originating from rabbit specimens in this investigation are demarcated in light blue. They did not form two distinct branches as initially hypothesized, with the rabbit-origin and human-origin strains each forming independent lineages. Notably, all the strains analyzed also carried additional antimicrobial resistance determinants, including *tet(A)* conferring tetracycline resistance, *folR* associated with sulfonamide resistance, and the multidrug efflux pump gene *mdf(A)*.

## 4 Discussion

Polymyxin, as a last-resort antibiotic (Mohapatra et al., 2021), has drawn the focus of global researchers since the emergence of the plasmid mediated resistance gene *mcr-1* (Liu et al., 2016). Rabbit meat is an important source of protein in Sichuan province and has become one of the most commonly consumed meats in the local diet. However, few studies have specifically focused on AMR issues in rabbit farms in China, despite evidence from prior studies indicating that a significant amount of antimicrobials is consumed during the meat rabbit breeding process (Silva et al., 2024). Consequently, there is an urgent need to establish AMR monitoring for rabbits, particularly in Sichuan province.

*Enterobacteriaceae* serve as critical vectors in the global dissemination of the mobile colistin resistance gene *mcr-1* (Xiaomin et al., 2020). In this study, we comprehensive investigated of AMR profiles across ten intensive meat rabbit farms. Four clinically relevant *Enterobacteriaceae* species were isolated from farm samples: *Klebsiella pneumoniae*, *Salmonella enterica*, *Enterobacter hormaechei*, and *Escherichia coli*. Although these pathogens have been extensively documented in human infections and livestock reservoirs (Lammie and Hughes, 2016; Elbediwi et al., 2020; Wyres et al., 2020), data from meat rabbit production systems remain strikingly sparse. Our study provides a reference and establishes a curated genomic BioProject (PRJNA1223317) for future mechanistic investigations into ARG transmission within rabbit farming systems. Furthermore, these data contribute to “One Health” surveillance strategies by highlighting the need to expand monitoring beyond conventional food-producing animals. Smaller-scale breeding operations may serve as overlooked reservoirs of antimicrobial resistance determinants.

Among the isolates collected in this study, the majority were *E. coli* (83.6%, 61/73), and the predominant STs were ST328, ST224, and ST297. These STs differed significantly from *E. coli* isolates previously obtained from clinical patients, swine, poultry, and other animals in China (Aworh et al., 2021; Peng et al., 2022). Notably, *E. coli* ST328 was also reported to produce extended-spectrum beta-lactamases (Gruel et al., 2022) and is associated with atypical enteropathogenic *E. coli* (Xu et al., 2017). This indicated that the resistant *E. coli* strains isolated from rabbits may differ from those isolated from pigs, further indicating that smaller-scale breeding operations may constitute neglected reservoirs of antimicrobial resistance determinants. In addition, *E. coli* serotype O178:H7 was dominant. This serotype was previously identified in pathogenic strains isolated from food and humans (Prager et al., 2009; González et al., 2017), yet its prevalence on rabbit farms has received limited attention. This discovery provides additional insight into the epidemiological transmission of pathogenic *E. coli* between humans and animals.

The AST results showed that resistance rates for tetracyclines, quinolones, and sulfonamides exceeded 60%, and more than 86% of the isolates exhibited multidrug resistance. These findings further underscored the significance of addressing AMR in rabbits. Notably, the ciprofloxacin resistance rate reached 74%, which was significantly higher than the rate previously reported in *E. coli* in pigs in China (Peng et al., 2022), and even surpassed the resistance rates observed in *E. coli* isolates from hospitals in China (CHINET data) (Luo et al., 2024). This finding suggested that quinolone antimicrobials may have been extensively used in meat rabbit farming in Sichuan. Unfortunately, the antibiotic administration history of the rabbit farms in this study was unavailable, as the owners of the sampled farms were unwilling to disclose their antibiotic usage. This maybe limited the epidemiology data collection. The majority of studies commonly rely on farmer-administered questionnaires to collect such data, which can lead to inherent subjectivity in the resulting information. Many policies are formulated based on the conclusions of epidemiological studies, which may consequently contribute to a higher likelihood of irrational antimicrobial use and, in turn, accelerate the emergence and spread of AMR.

The most remarkable finding was that 35 isolates carried the *mcr-1.1* gene, and the whole-genome sequence analysis revealed that some *mcr-1.1* genes were located on plasmids. Plasmids that harbor IncX4 and IncI2 plasmid replicons are well-documented vectors of interspecies transmission between animals and humans (Liu et al., 2018; Binsker et al., 2023). Of particular interest, 82.9% (29/35) of *mcr-1* positive isolates in our cohort carried IncI2-type plasmid replicons, suggesting a potential host-specific predominance of this replicon type in rabbit-derived strains. This replicon preference may indicate an elevated transmission risk of *mcr-1* from rabbit reservoirs to human populations. Current epidemiological data on the *mcr-1* prevalence in Chinese rabbit populations remain limited. We conducted a search for domestic relevant literature in the PubMed database using the keywords “*mcr-1*” AND “rabbits”. Only one previous study was found to be of reference value, which reported a 14.6% (8/55) positivity rate of *mcr-1* among *E. coli* isolates derived from rabbits in Shandong province (Wang et al., 2020a). Strikingly, our findings demonstrate a three-fold higher prevalence (48%, 35/73) in Sichuan province, highlighting significant regional disparities that urgently require scientific attention. This high prevalence identifies rabbit farms as a potentially critical reservoir for *mcr-1* persistence and dissemination. Since the plasmid mediated *mcr-1* gene was first reported, the use of polymyxin in livestock as an antibacterial growth promoting agent has been prohibited in China. Although surveillance data have shown a gradual decline in colistin resistance rates (Wang et al., 2020b), polymyxin resistance continues to persist, posing a potential threat to public health. This persistence emphasizes the imperative to implement sustained monitoring of polymyxin resistance patterns coupled with enhanced biosecurity measures in animal production systems.

Isolates positive for the *mcr-1.1* gene, including *Klebsiella pneumoniae*, *Salmonella enterica*, *Enterobacter hormaechei*, and *Escherichia coli* have demonstrated a robust capacity for horizontal gene transfer to recipient strains. The genetic environment of *mcr-1* has been elucidated, in which the *mcr-1* gene is flanked by *PAP2* and relaxase-coding genes. Relaxases are crucial in the horizontal transfer of ARGs (Valenzuela-Gómez et al., 2023). *PAP2* was frequently reported to be located in close proximity to the mobile *mcr-1* gene and may specifically participate in the *mcr-1.1* conjugation process (Peng et al., 2019). In addition, four functional clusters associated with mobile genetic elements were observed on the plasmid, likely explaining its strong capacity for horizontal transfer. The plasmid homology analysis of the *mcr-1* carrying plasmids performed in this study revealed a similarity to plasmids identified in nosocomial infections and livestock. The hosts of these plasmids include swine, cattle, and humans. Furthermore, the single nucleotide polymorphism phylogenetic analysis showed that the rabbit-derived strains did not separate from human-originating strains, instead, they exhibited close relatedness to each other and harbored numerous ARGs. However, whether these strains can be transmitted between rabbits and humans remains to be substantiated with additional evidence. Nonetheless, it was confirmed that rabbit farming exhibits a high prevalence of *mcr-1* and other ARGs. According to our best knowledge, there are few reports of any research indicating that rabbit-originating *mcr-1* positive enterobacteria can directly spread to humans.

This study has several limitations that should be acknowledged. First, the epidemiological investigation was constrained by a relatively limited sample size collected from a specific geographic region. Future studies would benefit from the inclusion of a larger, geographically diverse sample cohort to enhance the statistical power and generalizability of the findings. While our epidemiological investigation identified rabbit farms as potential reservoirs for *mcr-1*-positive *Enterobacteriaceae*, the experimental design did not provide sufficient evidence to confirm direct transfer of *mcr-1* from rabbits to humans. Notably, existing evidence from foodborne pathogen surveillance systems suggests that ARGs can traverse ecological boundaries through food supply chains, as demonstrated in many agricultural food production systems (Tiedje et al., 2023). However, the zoonotic transmission dynamics of *mcr-1*-harboring strains in lagomorph-derived food products remain uncharacterized. Systematic surveillance is needed to confirm these potential transmission routes. This warrants further molecular epidemiological investigation, including whole-genome sequencing of bacterial isolates across the farm-to-fork continuum and exposure risk assessment in human populations.

While rabbit farming is a significant part of the Chinese food industry, the overuse of antibiotics, particularly polymyxins, poses a serious threat to both animal and human health. Although there are valuable tools for monitoring antibiotic resistance, the lack of systematic testing in the rabbit farming sector undermines efforts to tackle this problem. Our systematic analysis of Sichuan province meat rabbit farms revealed widespread colonization by clinically relevant *Enterobacteriaceae* (*Klebsiella pneumoniae, Salmonella enterica, Enterobacter hormaechei*, and *Escherichia coli*), with 48% of isolates harboring the mobile colistin resistance gene *mcr-1*. Notably, the genomic characterization and *in vitro* conjugation experiments confirmed the plasmid-mediated transfer of *mcr-1* among these strains, with phylogenetic clustering patterns suggesting potential zoonotic transmission pathways between livestock reservoirs and humans. The results highlight that smaller-scale breeding operations may constitute neglected reservoirs of antimicrobial resistance determinants that require systematic assessment.

## Data Availability

The datasets presented in this study can be found in online repositories. The names of the repository/repositories and accession number(s) can be found in the article/supplementary material.

## Supplementary material

Supplementary Figure 1 The proportion of strains classified as multidrug-resistant, in which isolates exhibited resistance to three or more antimicrobial agents. The percentage of isolates resistant to multiple drugs is shown in various colors.

Supplementary Figure 2 The heat map illustrates the presence or absence of virulence factor genes in isolates; red cells indicate gene presence, while blue cells represent gene absence.

Supplementary Figure 3 Map and homology analysis of the *mcr-1*-positive plasmid pEc-CD45-5. **A.** The map of pEc-CD45–5 is presented, with the rings from inside to outside representing GC content, GC skew, coding sequences, integration and excision regions, replication, recombination, and repair functions, transfer mechanisms, and stability, transfer, and defense modules. **B.** Homology analysis of the pEc-CD45–5 plasmid is shown, with the rings from inside to outside representing pEc-CD45-5, pPSS-08-2_3(origin: human; geographic location: Ecuador; size: 60,961 bp; accession number: NZ_AP027682.1), pPSS-16_2 (origin: human; geographic location: Ecuador; size: 60,960 bp; accession number: NZ_AP027715.1), pHLJ109-70 (origin: chicken; geographic location: China; size: 61,023 bp; accession number: NZ_MN232201.1), pHLJ111-18 (origin: chicken; geographic location: China; size: 60,962 bp; accession number: NZ_MN232205.1), pHLJ111-5 (origin: chicken; geographic location: China; size: 61,094 bp; accession number: NZ_MN232208.1), and pSC111 (origin: human; geographic location: China; size: 60,960 bp; accession number: NZ_MZ277864.1). The outermost red ring represents coding genes.

Supplementary Figure 4 Amplification of the *mcr-1* gene in donors, recipients, and transconjugants. Lane 1 contains a DNA ladder (2000 bp). Lanes 2–6 represent the donor strains (Ec-JB2, Ec-CD45, Ec-A21, Ec-A24, Ec-A29, respectively). Lane 7 corresponds to the recipient strain (*E. coli* J53). The remaining lanes correspond to transconjugants derived from the specified strains: lanes 8–10, Ec-JB2; lanes 11–13, Ec-CD45; lanes 14–16, Ec-A21; lanes 17–19, Ec-A24; and lanes 20–22, Ec-A29. Lane 23 serves as the negative control.

Supplementary Table 1 The minimal inhibitory concentration value and resistance profile of isolates.

Supplementary Table 2 The sequence types of *Enterobacteria* isolates.

Supplementary Table 3 Serotype prediction results for the isolated *E. coli* strains.

Supplementary Table 4 Phylogroup prediction results for the isolated *E. coli* strains.

Supplementary Table 5 Antimicrobial resistance genes of the isolated strains annotated via the ResFinder database.

Supplementary Table 6 Antimicrobial resistance genes of the isolated strains annotated via the Comprehensive Antibiotic Resistance Database.

Supplementary Table 7 The results of plasmid replicon types from isolates.

Supplementary Table 8 Virulence factor genes of the isolated strains annotated via Virulence Factor Database.

Supplementary Table 9 The conjugation transfer frequency of Ec-A21, Ec-A24, Ec-A29, Ec-CD45 and Ec-JB2.
