# Supplementary material for: Antimicrobial resistance of Enterobacteriaceae in rabbit farms: an underestimated reservoir harboring mcr-1.1
Source: Front Cell Infect Microbiol. 2025 Oct 1;15:1663852. doi: 10.3389/fcimb.2025.1663852 (PMC12521101; doi:10.3389/fcimb.2025.1663852)
Supplement: Supplementary Figure 1 — The proportion of strains classified as multidrug-resistant, in which isolates exhibited resistance to three or more antimicrobial agents. The percentage of isolates resistant to multiple drugs is shown in various colors. [file DataSheet1.docx]

Supplementary Material

# Supplementary Figures and Tables

## Supplementary Figures


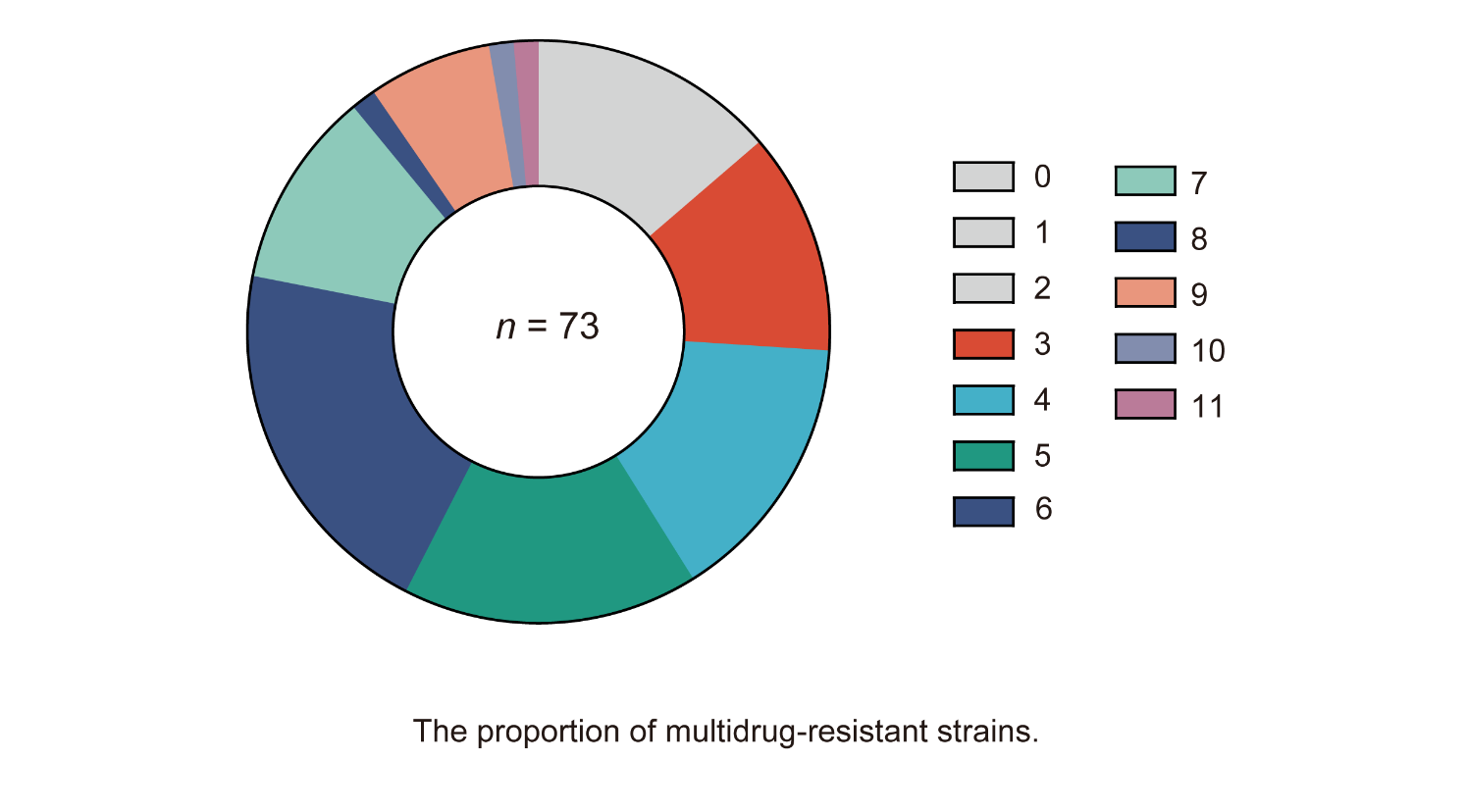


Supplementary Figure S1. The proportion of strains classified as multidrug-resistant, in which isolates exhibited resistance to three or more antimicrobial agents. The percentage of isolates resistant to multiple drugs is shown in various colors.


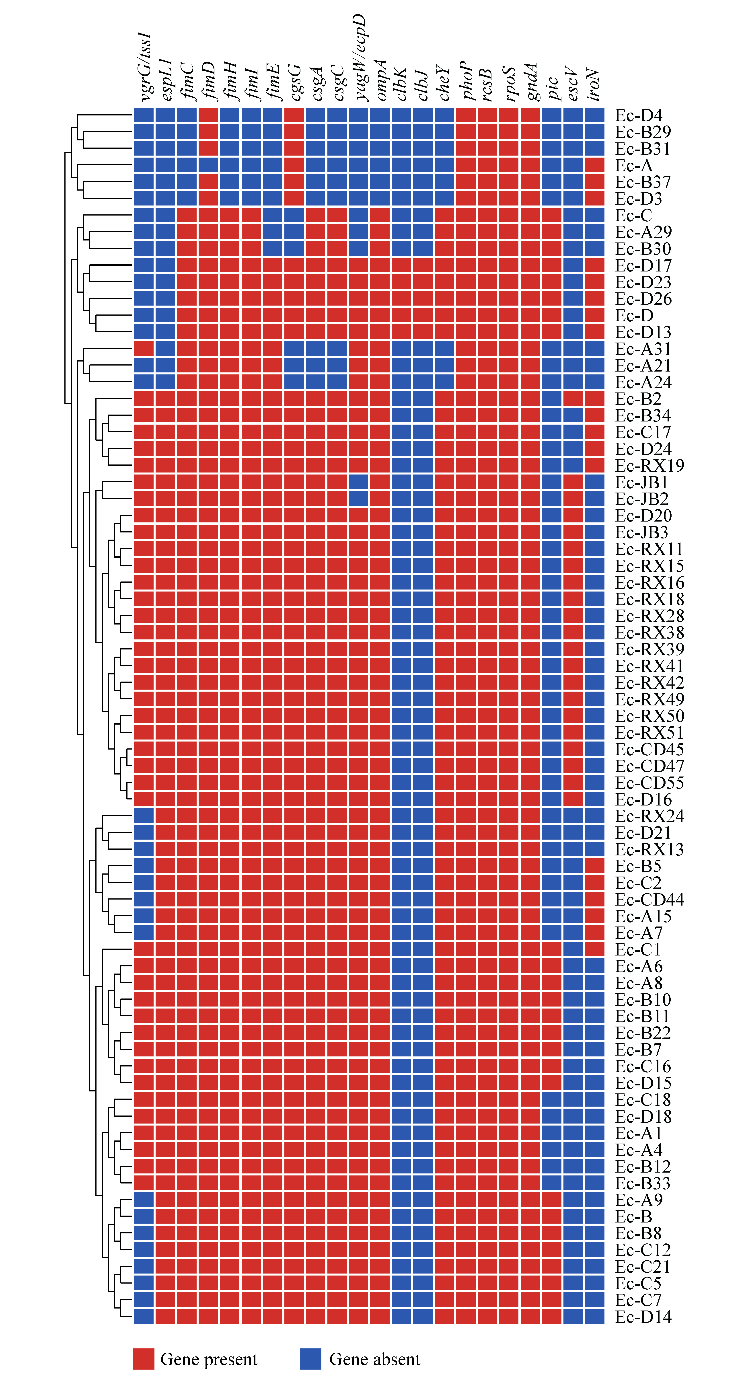


**Supplementary Figure S2.** The heat map illustrates the presence or absence of virulence factor genes in isolates; red cells indicate gene presence, while blue cells represent gene absence.


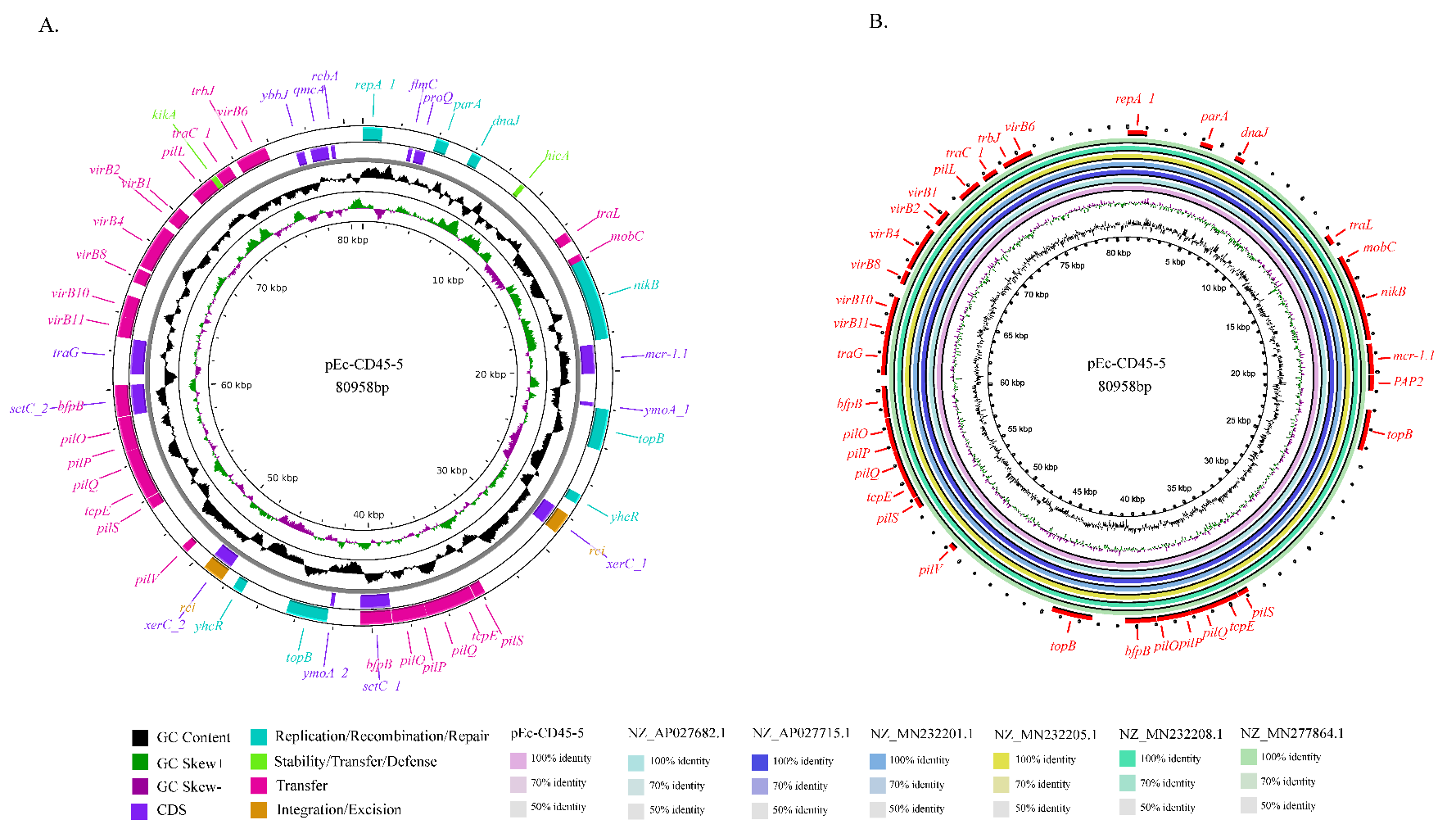
**Supplementary Figure S3.** Map and homology analysis of the *mcr-1*-positive plasmid pEc-CD45-5. **A.** The map of pEc-CD45-5 is presented, with the rings from inside to outside representing GC content, GC skew, coding sequences, integration and excision regions, replication, recombination, and repair functions, transfer mechanisms, and stability, transfer, and defense modules. **B.** Homology analysis of the pEc-CD45-5 plasmid is shown, with the rings from inside to outside representing pEc-CD45-5, pPSS-08-2_3 (origin: human; geographic location: Ecuador; size: 60,961 bp; accession number: NZ_AP027682.1), pPSS-16_2 (origin: human; geographic location: Ecuador; size: 60,960 bp; accession number: NZ_AP027715.1), pHLJ109-70 (origin: chicken; geographic location: China; size: 61,023 bp; accession number: NZ_MN232201.1), pHLJ111-18 (origin: chicken; geographic location: China; size: 60,962 bp; accession number: NZ_MN232205.1), pHLJ111-5 (origin: chicken; geographic location: China; size: 61,094 bp; accession number: NZ_MN232208.1), and pSC111 (origin: human; geographic location: China; size: 60,960 bp; accession number: NZ_MZ277864.1). The outermost red ring represents coding genes.


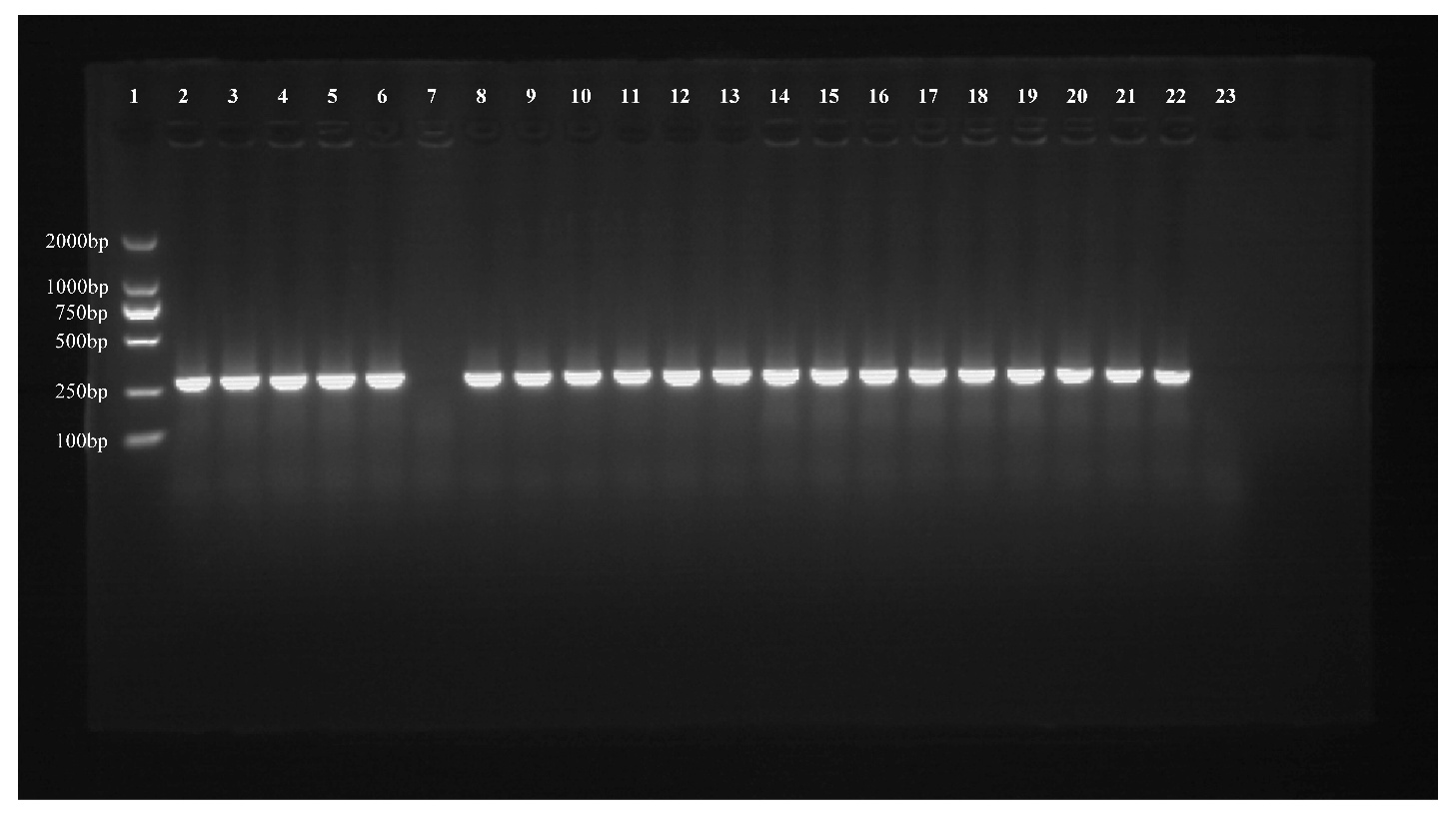
**Supplementary Figure S4.** Amplification of the *mcr-1* gene in donors, recipients, and transconjugants. Lane 1 contains a DNA ladder (2000 bp). Lanes 2–6 represent the donor strains (Ec-JB2, Ec-CD45, Ec-A21, Ec-A24, Ec-A29, respectively). Lane 7 corresponds to the recipient strain (*E. coli* J53). The remaining lanes correspond to transconjugants derived from the specified strains: lanes 8–10, Ec-JB2; lanes 11–13, Ec-CD45; lanes 14–16, Ec-A21; lanes 17–19, Ec-A24; and lanes 20–22, Ec-A29. Lane 23 serves as the negative control.

## Supplementary Tables

**Table S1.** The minimal inhibitory concentration value and resistance profile of isolates.

**Table S2.** The sequence types of *Enterobacteria* isolates.

**Table S3.** Serotype prediction results for the isolated *E. coli* strains.

**Table S4.** Phylogroup prediction results for the isolated *E. coli* strains.

**Table S5.** Antimicrobial resistance genes of the isolated strains annotated via the ResFinder database.

**Table S6.** Antimicrobial resistance genes of the isolated strains annotated via the Comprehensive Antibiotic Resistance Database.

**Table S7.** The results of plasmid replicon types from isolates.

**Table S8.** Virulence factor genes of the isolated strains annotated via Virulence Factor Database.

**Table S9.** The conjugation transfer frequency of Ec-A21, Ec-A24, Ec-A29, Ec-CD45 and Ec-JB2.
